# Automatic derivation of an MR-PET image-based input function for quantification of 18F-FET

**DOI:** 10.1186/2197-7364-2-S1-A27

**Published:** 2015-05-18

**Authors:** Nuno Andre da Silva, Liliana Caldeira, Hans Herzog, Lutz Tellmann, Christian Filss, Karl-Josef Langen, Jon Shah

**Affiliations:** Institute of Neuroscience and Medicine - 4, Forschungszentrum Jülich, Jülich, Germany

Fully quantitative PET data requires an input function (IF) for kinetic modelling that is often acquired via arterial blood sampling. Here, an automatic method to obtain an image-derived input function (IDIF) using MR-PET data is presented and several partial volume correction methods (PVC) are evaluated. Data from three tumour patients were acquired in a 3T MRBrainPET. A bolus of 3MBq/Kg of body weight of 18F-FET was administrated to each subject and a dynamic PET scan (dPET) was performed during 60min. PET data were reconstructed in 23 frames with variable frame length using OP-OSEM (32it, 4sub) including all the corrections. An MPRAGE scan was acquired (TE/TR/TI = 3/2250/900 ms, FA = 9°). Five venous blood samples (VBS) were drawn at the later times later times each 10min. To estimate an IDIF, internal carotid arteries were segmented automatically and these regions were transferred to dPET. After, 4 post reconstruction PVC were applied and the impact of a scaling factor based on a single VBS was also evaluated based on the area under the curve (AUC). All the tested PVC methods resulted in an under estimation of the AUC at later frames which was mitigated after scaling with a VBS at 50 min. In the earlier frames the different PVC resulted in different AUC, which were not possible to validate. The fully automated procedure presented allows one to obtain an IDIF without user interaction. Nevertheless, the initial findings regarding the PVC require further validation with a larger data set.

